# Supplementary figures and images for: Increased serum caspase-1 in adult-onset Still’s disease
Source: PLoS One. 2024 Jul 29;19(7):e0307908. doi: 10.1371/journal.pone.0307908 (PMC11285953; doi:10.1371/journal.pone.0307908)

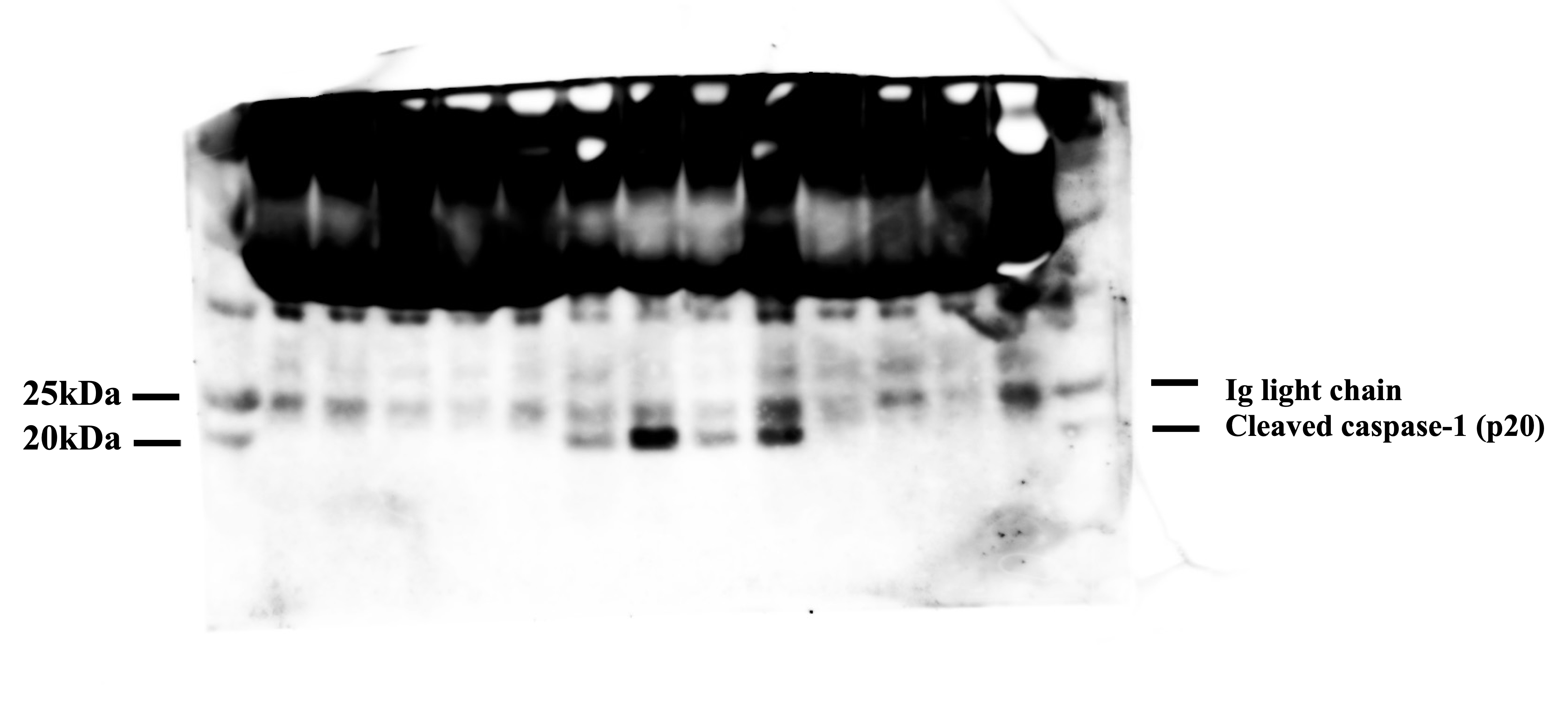

Supplement: S1 Fig — The cleaved caspase-1 band (p20) was detected in serum from patients with AOSD under reducing conditions (20 kDa). IgG light chain was also detected (25 kDa). (TIFF) [file pone.0307908.s001.tiff]

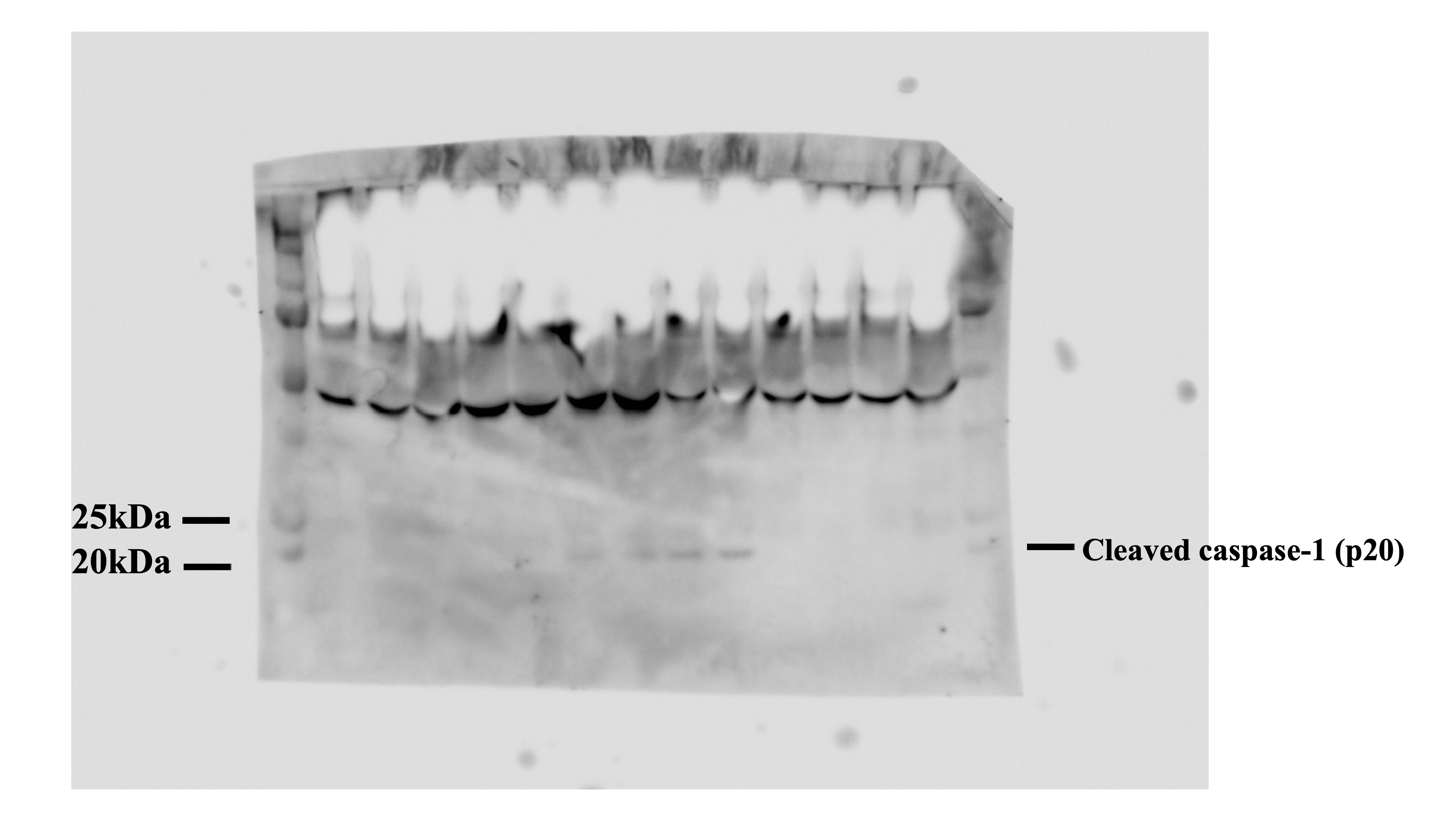

Supplement: S2 Fig — After IgG removal by protein G beads, cleaved caspase-1 was detected (20 kDa). (TIFF) [file pone.0307908.s002.tiff]

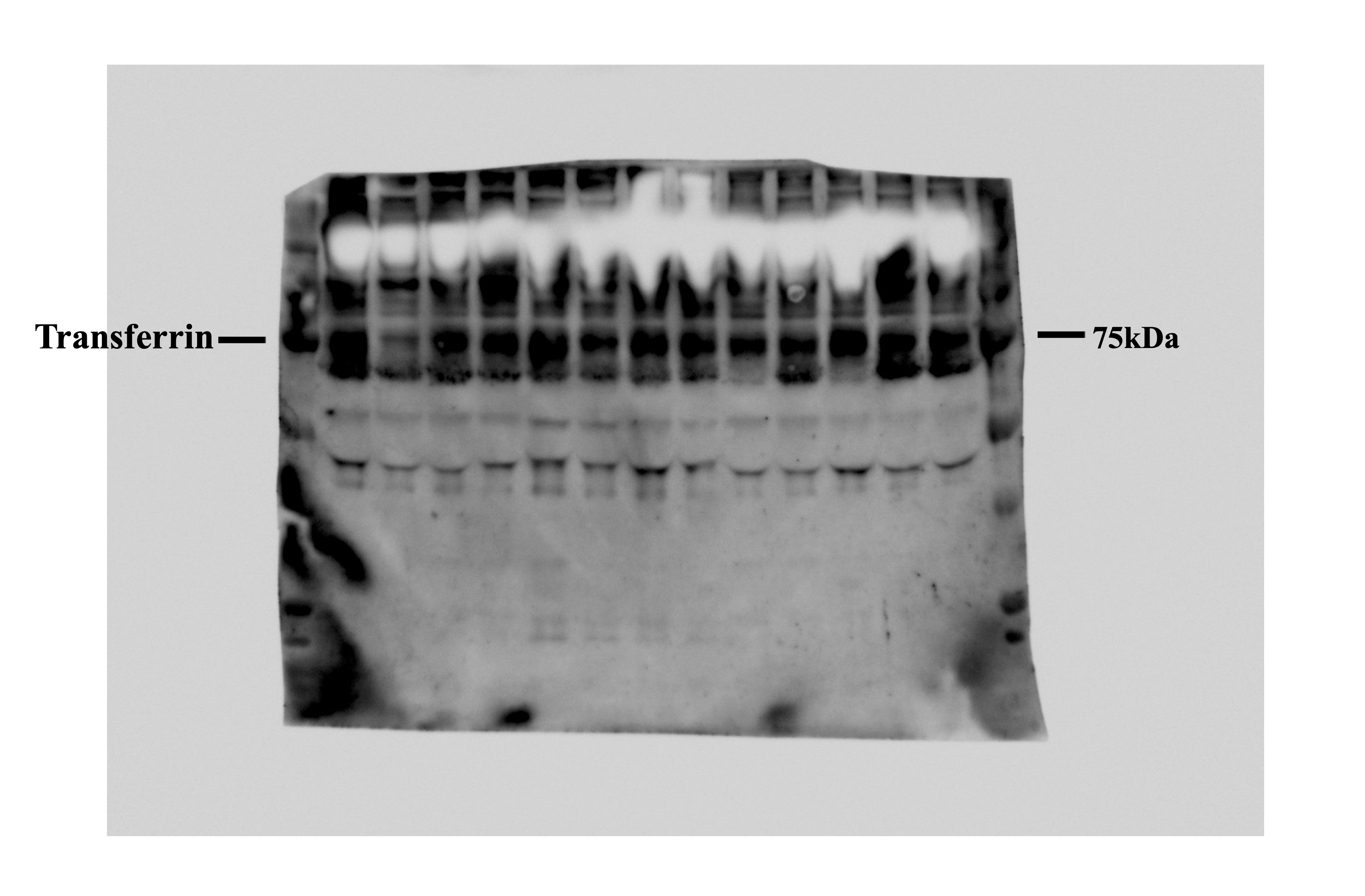

Supplement: S3 Fig — Serum transferrin was detected as the internal control to the same extent in untreated and treated patients with AOSD. (TIFF) [file pone.0307908.s003.tiff]
