# Supplementary material for: Increased serum caspase-1 in adult-onset Still’s disease
Source: PLoS One. 2024 Jul 29;19(7):e0307908. doi: 10.1371/journal.pone.0307908 (PMC11285953; doi:10.1371/journal.pone.0307908)
Supplement: S1 Table — (DOCX) [file pone.0307908.s004.docx]

Baseline characteristics of 66 Japanese patients with RA

| Characteristics Value | |
| --- | --- |
| Age (years), median (IQR) | 67 (62-74) |
| Female, n (%) | 39 (59.0) |
| Methotrexate, n (%) | 26 (39.4) |
| Biologics, n (%) | 17 (25.8) |
| DAS28-CRP, median (IQR) | 2.7 (2.2-3.6) |
| DAS28-ESR, median (IQR) | 3.4 (3.0-4.0) |
| CRP=C reactive protein, DAS28=Disease Activity Score, ESR=erythrocyte sedimentation rate, IQR=interquartile range. | |

Baseline characteristics of 36 HCs

| Characteristics Value | |
| --- | --- |
| Age (years), median (IQR) | 41 (28-52) |
| Female, n (%) | 39 (59.0) |
